# Transnational online education in biochemistry during and after the COVID-19 pandemic in Binzhou Medical University: challenges, strategies and outcome

**DOI:** 10.1186/s12909-023-04263-8

**Published:** 2023-04-20

**Authors:** Maxwell Ahiafor, Yanni Li, Xiaodan Zhang, Qun Ren

**Affiliations:** Department of Teaching and Scientific Research, School of International Studies, Shandong Province, Binzhou Medical University, 346 Guanhai Road, Laishan District, Yantai City, P.R. China

**Keywords:** International students, Transnational online education, DingTalk, Biochemistry, COVID-19

## Abstract

**Background:**

Due to the global outbreak of the COVID-19 epidemic, schools were forced to shift teaching from face-face to online. During this period, a large number of studies on how to better carry out online teaching emerged. However, these studies were basically conducted with domestic students as teaching objects. The research on transnational online education conducted by overseas students is very limited.

**Methods:**

We first conducted a questionnaire survey on the obstacles of transnational online learning of 64 international students from our school who were staying abroad at the beginning of the fall semester of 2020, analyzed the results using the two-tailed student’s t-test and changed the teaching platform accordingly and compared the results of the biochemistry exams conducted for 2018 spring class with those of 2018 fall class and the 2019 fall class, so as to verify the superiority of the DingTalk as a transnational online education platform.

**Results:**

The results showed that the main difficulties of overseas students in transnational online learning are poor network conditions and time difference. By using DingTalk as an online teaching platform, these difficulties were overcome. In the spring class of 2018, the results of online study students’ biochemistry were significantly lower than those of students in face-face study (*t-test, p* = *0.01*). However, after the switch to the DingTalk platform, online students’ results in the 2018 fall class (*t-test, p* = *0.35*) and the 2019 fall class (*t-test, p* = *0.7*) were equivalent to the academic performance of face-face students.

**Conclusion:**

Our exploration and application of DingTalk software in transnational online education successfully solved the dilemma of overseas students’ online learning, and provided a feasible method to guarantee the efficacy of online teaching.

**Supplementary Information:**

The online version contains supplementary material available at 10.1186/s12909-023-04263-8.

## Introduction

In December 2019, the outbreak of the novel coronavirus disease 2019 (COVID-19) spread to a global pandemic [1]. Countries around the world have been forced to switch from classroom teaching to online teaching [2]. However, problems of online platform teaching are also profound. Examples include technical barriers, poor internet connectivity and lack of quality trainers [3, 4]. It has been more than 2 years since China started online teaching after the pandemic. Currently, the internationally popular teaching platforms include Google Classroom, Google Meet, Skype, Zoom, etc. [5]. But, unfortunately none of the above platforms can be registered by teachers in China. Therefore, it is more difficult for Chinese teachers to carry out transnational online teaching. Studies have reported that even if the above online teaching platforms could be used, various challenges will be encountered, with the assessment and evaluation of learners through technology being the main difficulty [6].

Binzhou Medical University (BMU) established in 1946, has trained more than 600 international students from over 32 countries since 2006. At the beginning of the pandemic, our school was also actively exploring teaching platforms suitable for transnational online teaching, but no systematic solution was formed in a short period of time, and teachers could only complete basic daily teaching for overseas students. The problem of online teaching was gradually exposed. It was difficult to track the learning status of overseas students in time, and it was difficult to guarantee the quality of teaching. After a difficult semester of online study, our school collected problems and researched solutions in a variety of ways, including questionnaires, literature review and experience exchange with similar schools [7–10]. After weighing the advantages and disadvantages of various popular online platforms in China, we decided to introduce DingTalk software as a unified educational platform for international students in our university.

DingTalk is the international version of the “dingding” software developed by Alibaba Group, which is widely used worldwide because of its advantages of supporting multiple languages and smooth live broadcasting [11]. At present, many colleges and universities have used the DingTalk platform to carry out online teaching for students, and have accumulated valuable experience at the same time [12, 13]. However, there is a relative lack of research on transnational online education guarantee by using this platform. Therefore, we relied on the great advantages of globalization, multi-terminals and free use of DingTalk software to expand it to the field of transnational online education, so as to guarantee the teaching operation of international students in our university. Biochemistry is a very important basic medical subject, which mainly studies the structure and function of various components in cells, such as proteins, sugars, lipids, nucleic acids and other biological macromolecules [14]. Since the fall semester of 2020, our school has used the DingTalk platform to conduct transnational online biochemistry teaching for overseas students. By July 2022, we had completed the teaching of biochemistry for students of 2018 fall class and 2019 fall class. This case study aims to share the authors’ research and practical experience on transnational online teaching after the outbreak of the pandemic, and how to choose the appropriate teaching platform to ensure the quality of online teaching as well as provide reference for future research.

## Methods

### Data collection

These data through the Questionnaire Star platform (https://www.wjx.cn/) to complete the questionnaire (Table 1 and Additional file 1) and DingTalk platform between at the beginning of the fall semester of 2020 and the end of the 2022 spring semester. The participants were 64 overseas students studying clinical medicine at Binzhou Medical College. The questionnaires were evaluated anonymously, and abstention was allowed for each statement. Acquisition of data was done with the consent of participants and approval from Binzhou Medical University Ethics Committee.

**Table 1:**
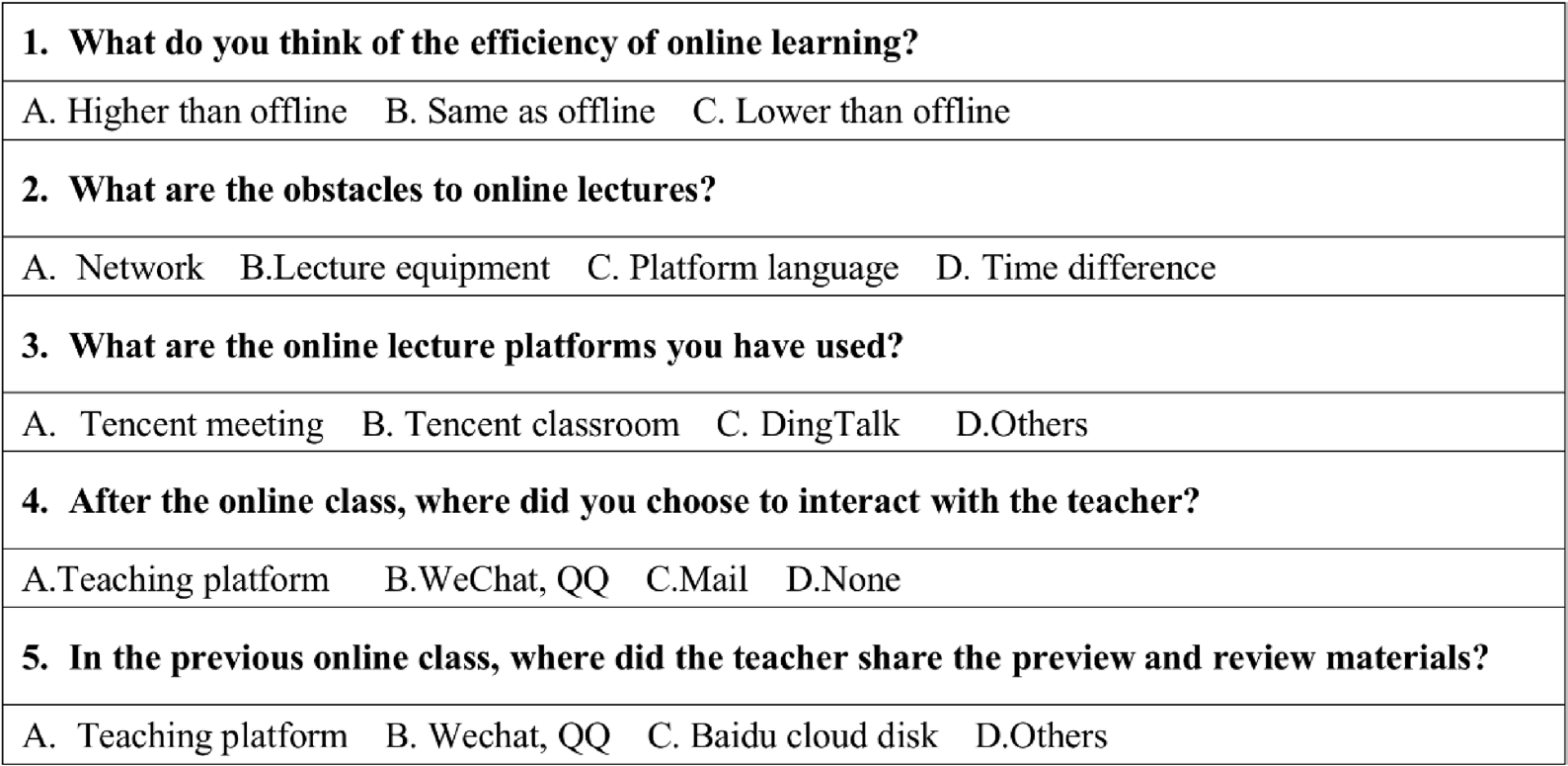
The details of questionnaire I

### Participants

We recruited 69 students for this survey. Five students were excluded because criteria such as active enrollment status, being registered for the course, etc.…, were not met. A total of 64 students were included in this survey. We used the International Student Information Management System (http://bzmc.liuguanbao.com) which was developed of Mr. Zhang Feng (zhangfeng@xmu.edu.cn) of Xiamen University to collect data on the country of origin of students (Table 2).

**Table 2:**
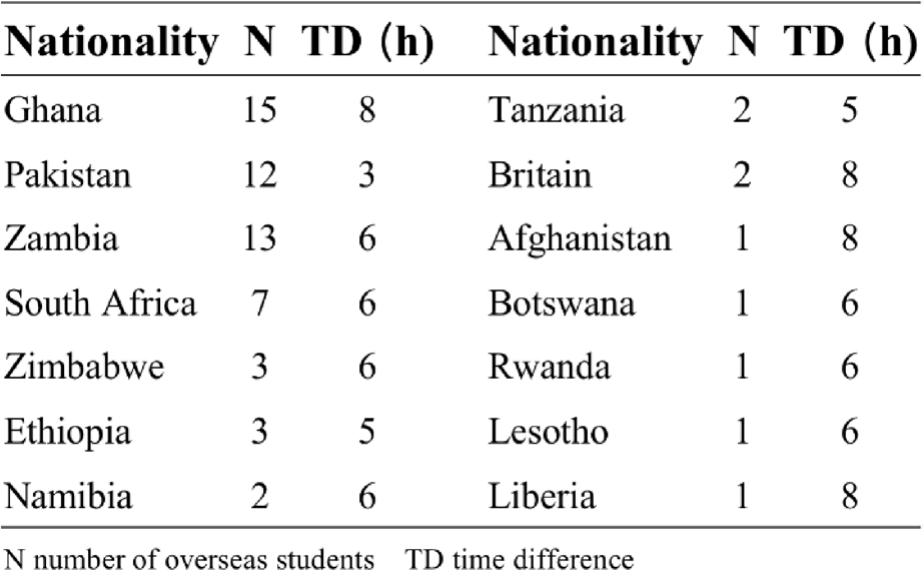
Time difference between the countries and local time of the 64 overseas students of our university and China

### Teaching analysis

Using DingTalk software (https://www.dingtalk.com) we carried out online teaching research, analysis of teaching data. The acquisition of such data was through the “class group” and “subject group” structure on DingTalk. The academic management office of our school created class/course groups on DingTalk then the respective students and teachers were added via invitation codes to their corresponding groups.

### Statistical analysis

Percentage of multiple choice options equals number of times the option is chosen divided by number of valid answers (a total of 64). The implication of this indicates the proportion of people who chose this option in the total number of people who filled in the option. So for multiple choice answers the percentages can add up to more than 100 percent. Two-tailed Student’s t-test was utilized for significance of differences between groups. Statistical was processed via SPSS 23.0 software and Graph-Pad Prism 8.0 (GraphPad Software, Inc., La Jolla, CA, USA). Probability values of *p* < 0.05 was considered as significant difference.

## Results

### Exploration of the barriers to international online teaching (challenges)

**Fig. 1:**
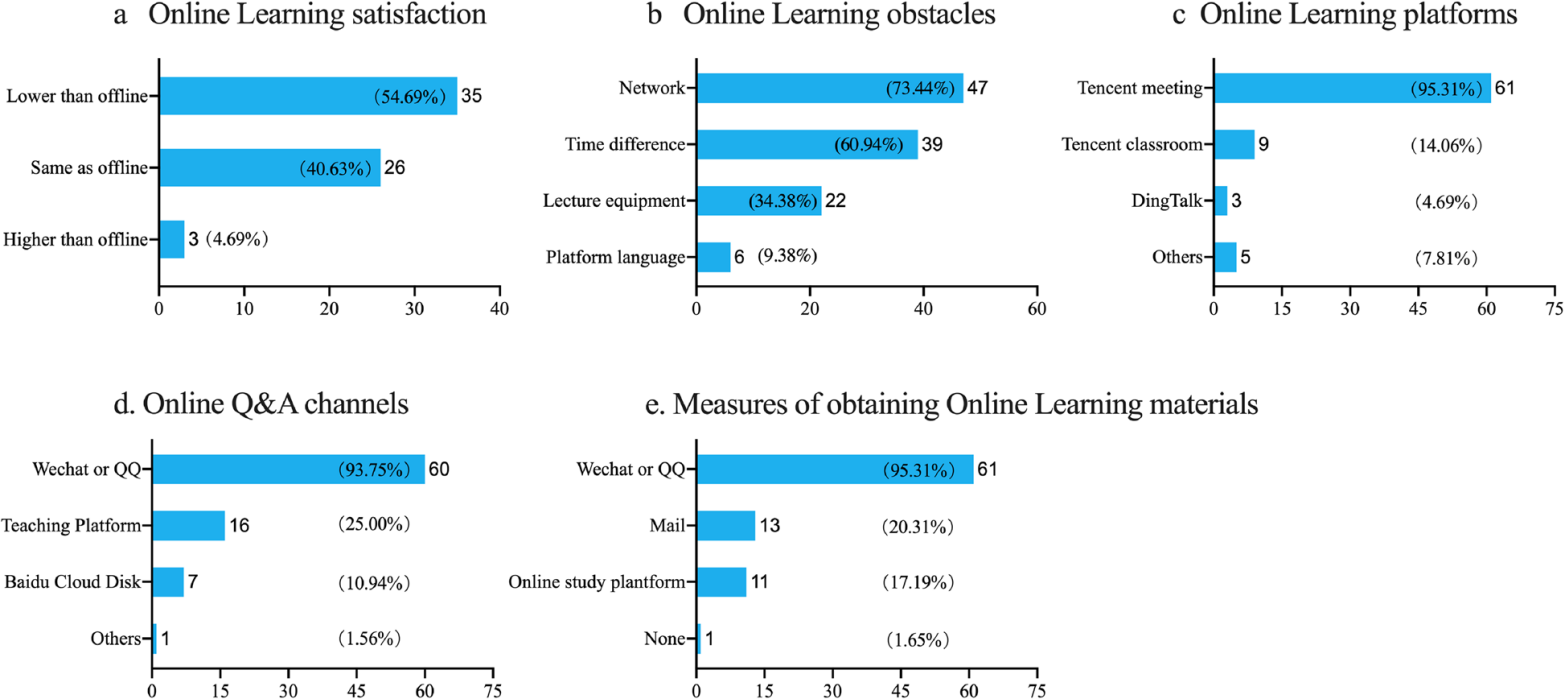
Exploration of the barriers to international online teaching. **a** Survey of online learning satisfaction. **b** Problems faced by overseas students in the early stage of transnational online teaching. **c** Platforms used by international students for online learning before Fall Semester 2020. **d**, **e** Ways international students interact with teachers and obtaining study materials before Fall Semester 2020. **b**-**e** multiple choice/choose any that apply

At the beginning of the fall semester of 2020, we conducted a questionnaire survey among overseas students who were unable to return to the university. The purpose was to obtain their feedback on the online teaching status of the university at that time after experiencing the online learning mode for one semester. A total of 64 valid questionnaires were collected, and the statistical results are shown in Fig. 1. We found that 54.69% of students said that online learning was less effective compared to offline learning (Fig. 1a). It can be seen that “network problems” (over 73%) and “local time difference” (over 60%) were the main challenges that troubled overseas students in the online semester (Fig. 1b). Furthermore, through the student status management system for overseas students, we investigated the regions where overseas students live and the time difference between local time and China. The data are up to 2020, and the statistical results are shown in Table 1. A total of 64 students stay abroad and almost all of them come from developing countries with some having relatively poor local network conditions and the insufficient power supply. In addition, there’s more than 50% of students with about 6 ~ 8 h time difference between the local time and China (Table 2). The above two factors are the main objective reasons that hinder transnational online education, resulting in a small number of online learners and poor classroom response. About online learning platforms, the number of people who use “Tencent Meeting” to courses study is the largest, accounting for more than 95% (Fig. 1c). However, more than 95% and 93% of students interact with teachers and obtain learning materials through Wechat and QQ, respectively (Fig. 1d, e). Through the survey of international students, we found that online learning was usually composed of online learning platforms (such as Tencent Meeting) and class groups using tencent’s messaging apps such as QQ and Wechat. Teachers broadcast their lectures through online platforms and send links to their classes via class groups. Consequently, we found that it’s very difficult for students to overcome the huge time difference and participate in live broadcast learning for a long time. Therefore, our teachers record and save the lessons synchronously, and then send them to the class groups. This method makes it difficult for us to monitor and review, in the long term, the lessons learned. Moreover, in the case of studying multiple subjects, the large number of lessons can easily lead to confusion, thus is difficult to ensure the quality of transnational online education.

### Establishing DingTalk as a unified platform for international students to use for online education (strategies)

Subject-based groups were built. Under the joint maintenance of teachers and student leaders, the group can realize a “one-stop” closed-loop mechanism of teaching and learning feedback, such as real-time class sessions, synchronous class recording, online Q&A, sharing of study materials, and teaching supervision. Live broadcast of courses in real time break international regional barriers; Cloud storage of synchronous recording can solve the problem of overseas students missing the teaching content due to network interruption and other emergencies; Online open Q&A on the same platform is conducive for the sharing of questions and answers, and avoids the dilemma of separating the teaching platform and Q&A platform.

**Fig. 2:**
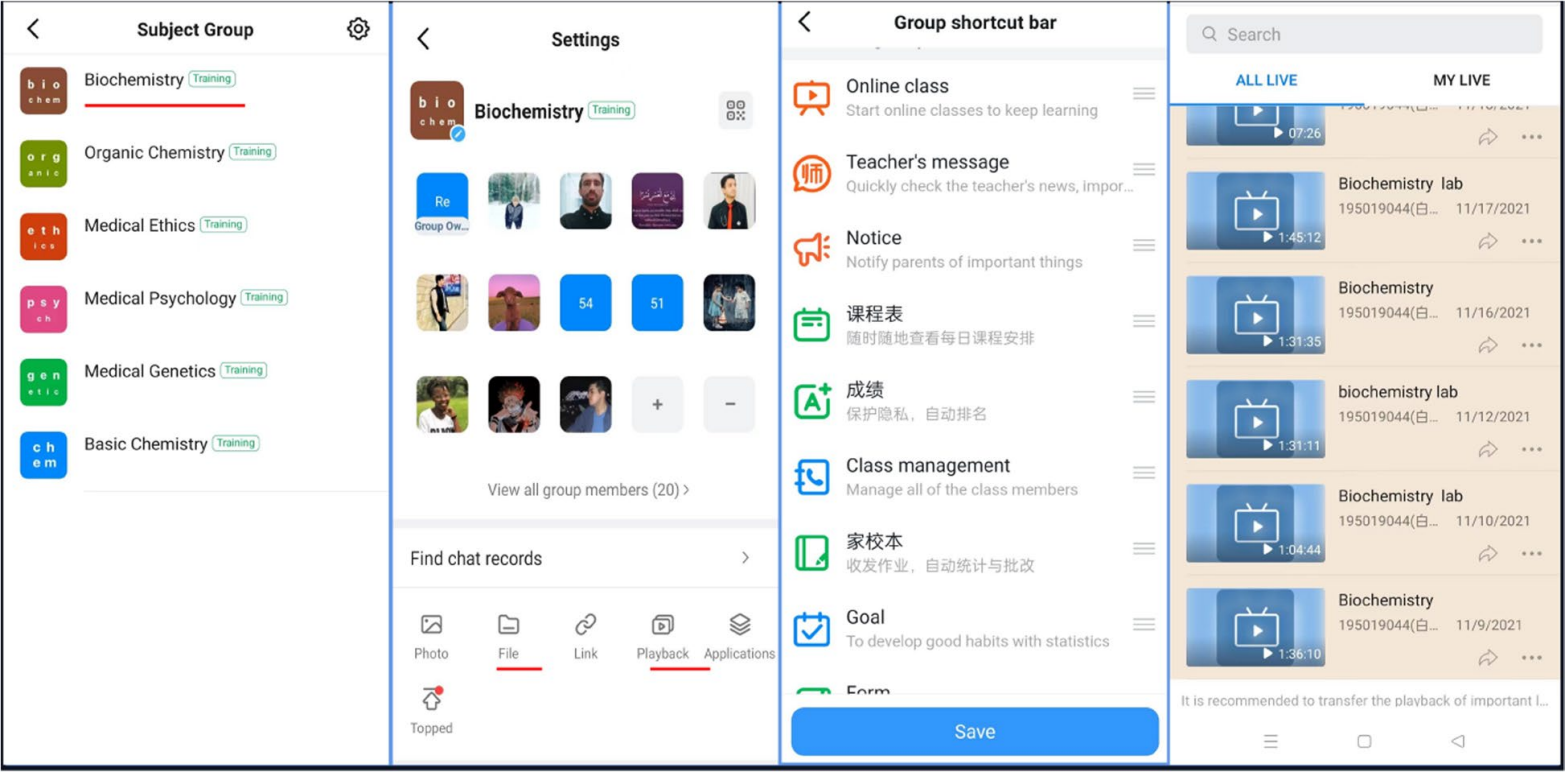
Establishing DingTalk as a unified platform for international students to use for online education. Curriculum: check the daily curriculum at any time (课程表: 随时查看每日课程安排); results: privacy protection, automatic ranking (成绩: 保护隐私、自动排名); Home school: sending and receiving homework, correcting homework, statistics (家校本: 收发作业、自动统计与批改)

As shown in Fig. 2, we set up the required “subject group” for different classes. No matter before class, during class or after class, students and teachers can discuss curriculum-related issues in the exclusive subject groups. During class, teachers can use this group to conduct live classes. Because DingTalk online live classes are automatically saved in the cloud, students who have time difference or lack of local network can learn through playback. All course initiation records will be stored in the cloud for at least 180 days, so students can review at any time during this period, greatly improving learning efficiency. In addition, teachers and administrators can output students’ learning records through the platform data to regularly supervise students who have not completed their studies, so as to ensure the quality of transnational online education.

### DingTalk facilitates biochemistry course international online teaching (Outcome)

**Fig. 3:**
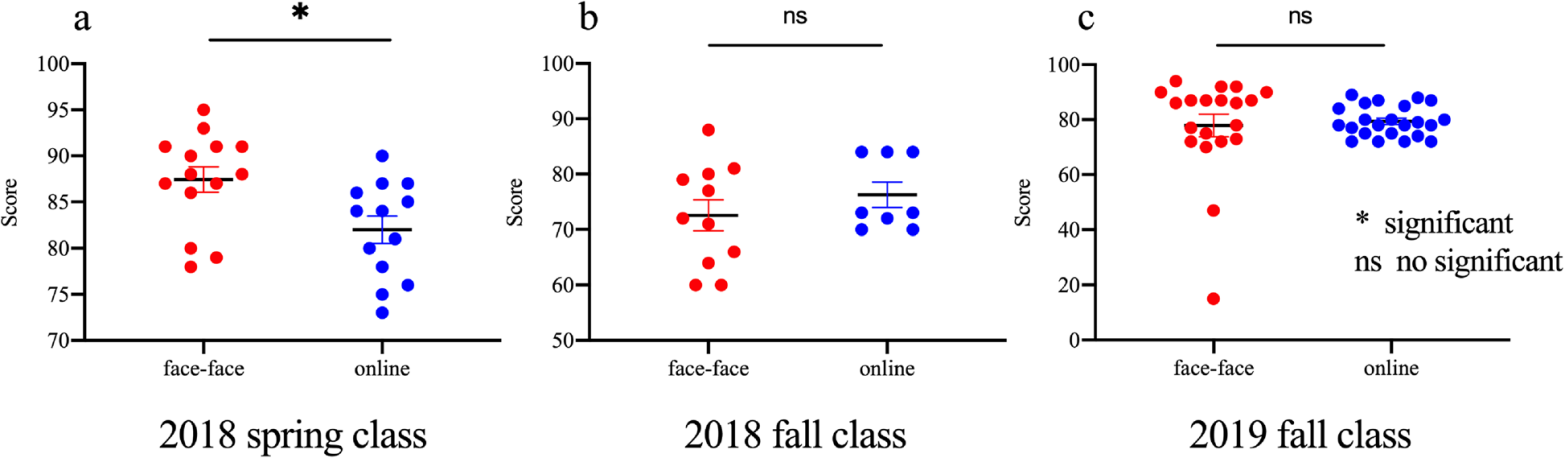
Comparison of online study and face-face study biochemistry scores before and after use of DingTalk platform for online teaching. **a** The biochemistry scores of all students in the 2018 spring class before using the DingTalk platform for teaching. **b**, **c** The biochemistry scores of all students in the 2018 fall class and 2019 fall class after using the DingTalk platform for teaching

On the basis of the comprehensive application of the DingTalk platform for international students in our school and to assess the effect of online teaching of the biochemistry course, we compared the biochemistry final exam scores of students in online learning group and face-face learning group in different classes (2018 spring Class, 2018 fall Class and 2019 fall Class). Of these three classes, 2018 spring class did not use DingTalk platform for online lessons, whereas 2018 fall and 2019 fall classes used DingTalk platform. The 2018 spring class had 27 international students who took this final examination, consisting of 13 in the online group and 14 in the face to face group. The average scores of the face-face group for biochemistry was 87.43 ± 1.39, while the average scores of online group was 82 ± 1.46. The scores of the face-face group’s students were statistically significantly higher than that of the online group (*p* = 0.01) (Fig. 3a). However, there was relatively little difference in exam scores between the online group (76.25 ± 2.30) and the face-face group (72.55 ± 2.80) of the fall class of 2018 (*p* = 0.35) and for fall class of 2019 (*p* = 0.7), online group (79.39 ± 1.16) and face-face group (72.85 ± 4.13) (Fig. 3b, c). Interestingly, we found that students in the online group had higher scores than those in the face-face group after using the DingTalk platform for lessons. Please see Table 3 for the specific statistical results.

**Table 3:**
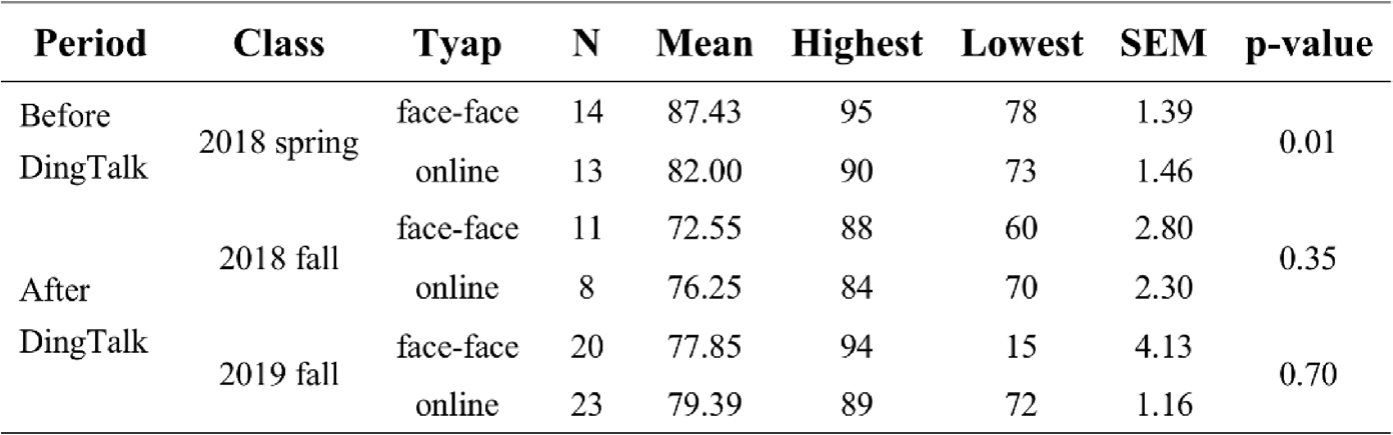
Statistical analysis of exam scores before and after the use DingTalk

**Fig. 4:**
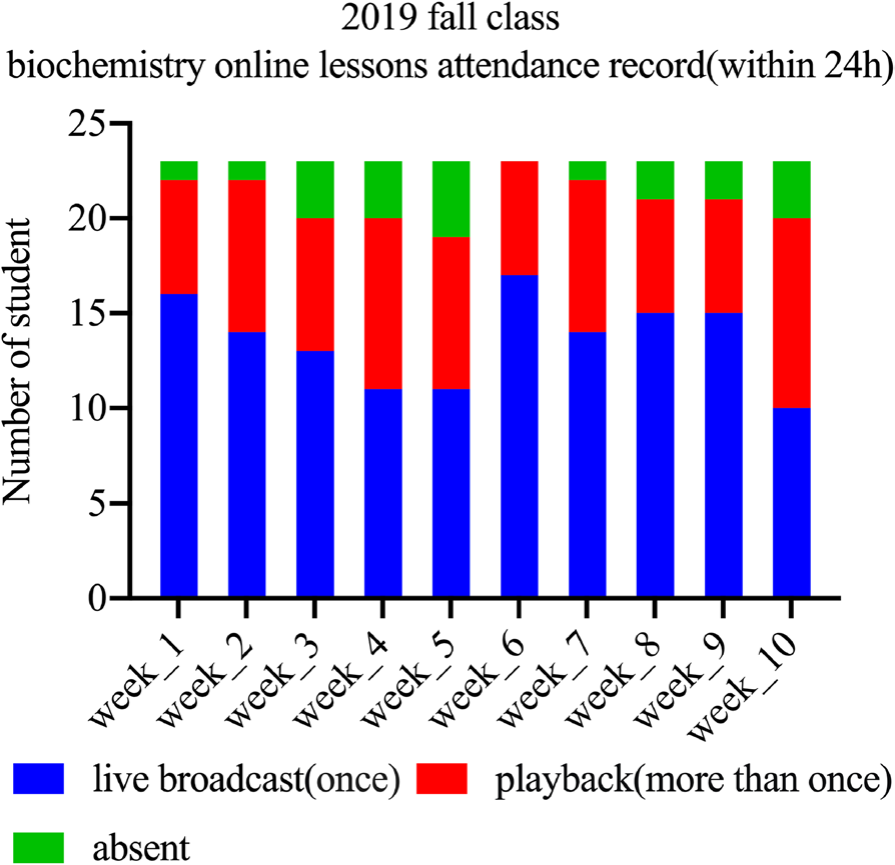
Study record within 24 h after the completion of biochemistry course for international students in 2019 fall class. Blue means the student has only learned once via live broadcast, Red means the student has learned it more than once via playback, and Green means the student has not participated either join the live broadcast or viewed playback within 24h

**Fig. 5:**
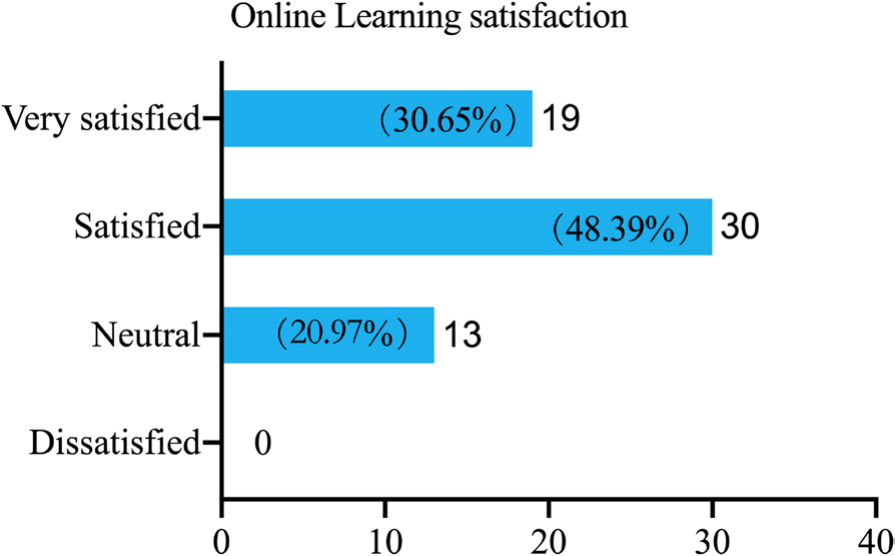
Online learning satisfaction after DingTalk unified transnational online education platform

We continuously tracked the lesson, learning styles and students’ feedback of biochemistry up to July 2022. We only show how the “playback” function in the platform contributes to teaching quality assurance, and the results are shown in Fig. 4. We can see that about 30% of the students repeated the study within 24 h (Table 4), probably because the students reviewed the course content. These data were also obtained from the DingTalk software database. Teaching administrators or class teachers can contact students in time base on this data to provide them with help if necessary. Also, our data (Fig. 5) from (Additional file 1: Table S1 question 4) shows that more that 79% of the students in the online group were satisfied with the use of DingTalk as a unified transnational online education platform. Of which 30.65% were very satisfied. It can be seen that in the face of two challenges that are difficult to overcome in transnational online education, education informatization helps to ensure the quality of teaching and provides opportunities for students who miss live broadcast sessions to learn again. This falls under the supervisory role of educational informatization in ensuring the quality of teaching.

**Table 4:**
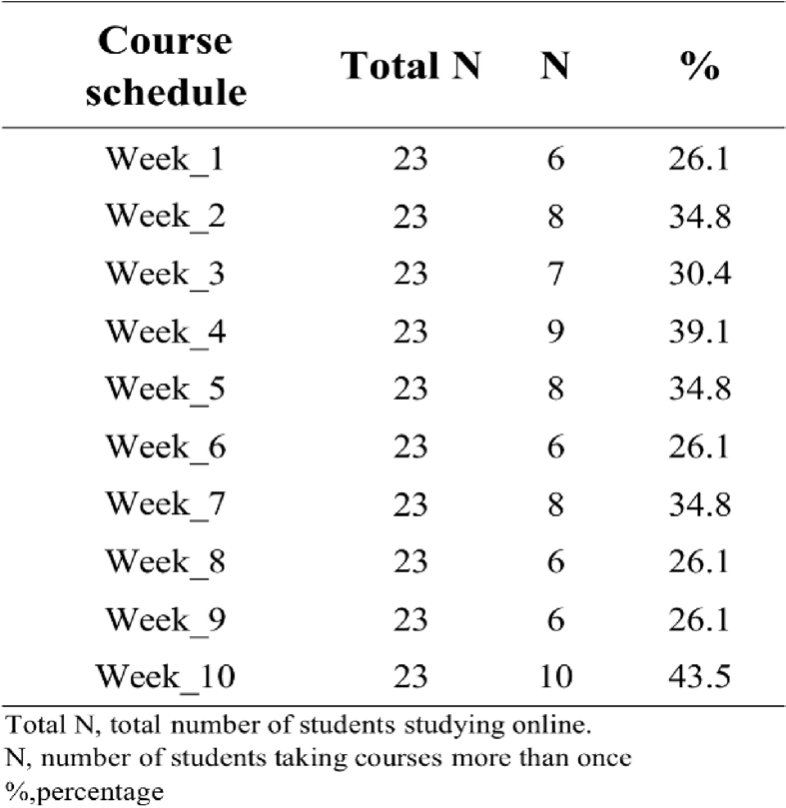
Weekly percentage of playback students in 2019 fall biochemistry class

## Discussion

In recent years, clinical medicine has become a popular major for foreign students to study in China [15]. With the increasing number of international students, the problem of education quality assurance has gradually emerged. At the end of 2019, the outbreak of the COVID-19 of the global pandemic caused the inability of a large number of international students to return to school, which is a severe challenge for international students’ education [16]. In the future and even for a long time, overseas students will conduct their studies through transnational online learning. Therefore, it is urgent to study how to guarantee the quality of education and teaching of international students [17]. In combination with the online teaching experience of international students in the early stage of the pandemic, we found the following difficulties in the teaching of overseas students: time difference in the region where learners live, prominent network problems, teachers’ use of recording classes, lack of classroom interaction, and the separation of teaching and Q&A platforms, making it difficult to get teaching feedback in time [18]. In view of the above prominent problems, we conducted an in-depth research through a variety of ways, integrated the online teaching platforms that have been successfully used in China, combined with the particularity of international students’ online learning, and balanced the advantages and disadvantages, then decided to use DingTalk as a unified teaching platform for international students. The real-time opening of broadcast of lessons in the Dingtalk software breaks international regional barriers; Compared to other online teaching platforms, Dingtalk has extra functions including automatic playback (up to 180 days after broadcast), class group formation, interactive sessions (before, during and after class), attendance checking and evaluation, online and offline access to shared materials, no expiration date on shared materials ie. ppt, documents, videos, audios, etc.… Students and teachers who join the group at a later time can view up to 100 messages prior to their time of joining the groups, a function other platforms do not possess. Cloud storage of synchronous recording can solve the problem of overseas students who miss the teaching content due to network interruption and other emergencies; Online open Q&A on the same platform is conducive for the sharing of questions and answers, and avoids the dilemma of separating the teaching platform and Q&A platform. In the process of using information technology to help our international education reform, we continue to accept feedback from students and teachers, and strive to solve the difficulties faced by transnational online education.

With the advent of the post-pandemic era, Chinese students have resumed to offline teaching as the domestic epidemic has leveled off. However, online learning is still the main way for overseas students to study while they cannot return to China due to prevention policy. With the progress of the epidemic, the previous “comprehensive online teaching” has shifted to the current “online + offline” hybrid teaching. For international students, how to guarantee the simultaneous teaching of domestic and overseas students is a challenge, but also an opportunity in the current information age. On a global scale, the use of DingTalk has enhanced the way online education is conducted. It has breached the gab between developed and developing nation in terms of knowledge acquisition through online teaching by providing a reliably efficient platform that isn’t limited by time difference, poor internet connection among others.

Information shared via this platform has no expiry date and playbacks can still be viewed up to 180 days after the live broadcast make it convenient for students in areas of poor network and huge time difference to get access materials and adequate course contents Therefore, our research on information-based international education will be a protracted battle.

## Limitations

This study only takes students from the author’s school as the research object. So this study may be limited in it’s global application.

## Conclusions

International online teaching has been carried out for more than 2 years, we deeply realize that the key to the success of online teaching is quality assurance. We believe that quality assurance can be divided into two aspects: assistance and supervision. First, how do we assist overseas students to complete online learning in the face of various difficulties such as time difference and internet connectivity problems; Second, how do teachers and administrators supervise students’ learning status and use the teaching platform to capture students’ learning data. In the above two aspects, we can use the DingTalk software to achieve results smoothly and help improve students’ exam scores as seen in the scores of the 2018 and 2019 fall classes. Combined with our practical experience in transnational online education, this study concludes that in the context of highly information-based education, the appropriate selection and efficient use of teaching and learning platforms is essential, just as a good job must first improve its tools.

## Supplementary Information

**Additional file 1: Table S1.** The details of questionnaire II.

## Data Availability

The datasets of this article are obtainable from the corresponding author on a reasonable request.

## Abbreviations

COVID-19: Coronavirus disease 2019
Q&A: Question and Answer
Wechat, QQ: Messaging and social media application software
Google Classroom, Google Meet, Skype, Zoom: Video meetings application software
