# Supplementary material for: Transnational online education in biochemistry during and after the COVID-19 pandemic in Binzhou Medical University: challenges, strategies and outcome
Source: BMC Med Educ. 2023 Apr 20;23:266. doi: 10.1186/s12909-023-04263-8 (PMC10115598; doi:10.1186/s12909-023-04263-8)
Supplement: Supplementary file 1 — Additional file 1: Table S1. The details of questionnaire II. [file 12909_2023_4263_MOESM1_ESM.doc]

**Table S1** The details of questionnaire II


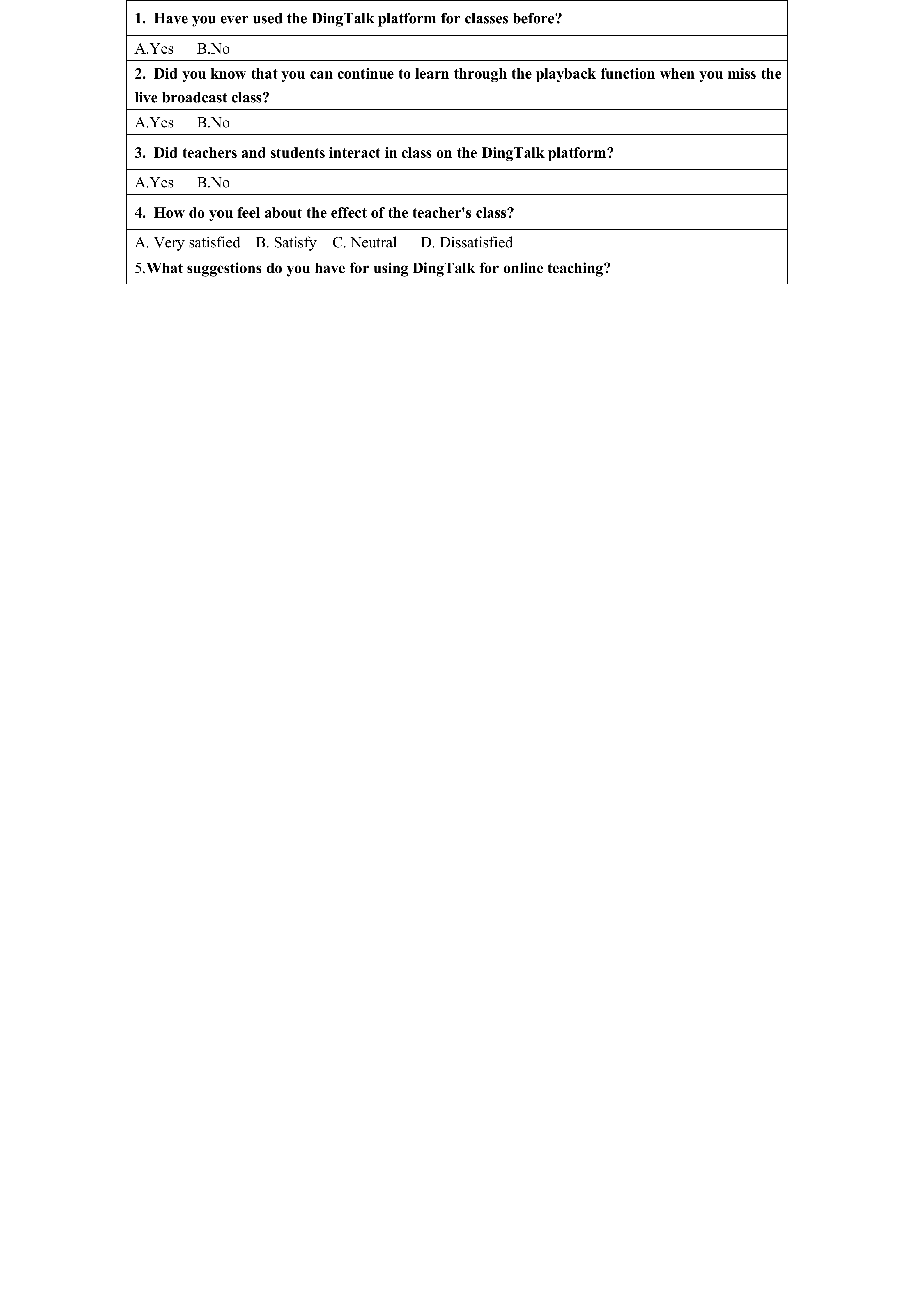


This table shows the contents of the anonymous questionnaire survey we issued to obtain students' feedback after conducting online teaching via DingTalk platform for at least one semester.It assesses students' knowledge of the additional functions of Dingtalk pertaining to online teaching and also students' satisfaction levels.
